# Compressive Failure and Dual-Defect Coupling Effects of Open-Hole Composite Laminates with Drilling-Induced Delamination

**DOI:** 10.3390/ma18122790

**Published:** 2025-06-13

**Authors:** Rui Zhu, Yonghui Liu, Xingyue Nie, Qingqing Xiao, Jingpu Liang, Dongfeng Cao

**Affiliations:** 1 School of Information and Mechanical and Electrical Engineering, College of Arts and Sciences, Yangtze University, Jingzhou 434020, China; ryan@wlxy.edu.cn (R.Z.); nxy99@wlxy.edu.cn (X.N.); xqq22@wlxy.edu.cn (Q.X.); umapyoi@wlxy.edu.cn (J.L.); 2 State Key Laboratory of Materials Synthesis and Processing, Wuhan University of Technology, Wuhan 430070, China

**Keywords:** compressive failure, dual-defect coupling, open-hole composite laminates, drilling-induced delamination

## Abstract

This study investigates the influence of drilling-induced delamination damage on the compressive mechanical behavior of open-hole carbon fiber-reinforced composite laminates and explores the failure mechanisms under dual-defect coupling effects. Specimens with circular delamination defects of varying sizes were fabricated by embedding polytetrafluoroethylene (PTFE) films during the layup process. Ultrasonic C-scan and digital image correlation (DIC) techniques were used to monitor delamination propagation and deformation behavior. A cohesive zone-based numerical model was developed and validated against experimental results to reveal the three-stage failure process in single-defect cases. The validated model was then used to analyze the coupling effects of dual defects (same side and opposite side). The results show that dual delamination defects significantly reduce the compressive load-bearing capacity of open-hole composite laminates. Specifically, same-side defects exhibit a failure mode similar to single-defect structures, while opposite-side defects display a unique failure behavior characterized by dual-crack propagation, further reducing the compressive load-bearing capacity.

## 1. Introduction

Carbon fiber-reinforced plastic (CFRP) is widely used in aerospace, aviation, and automotive industries for its high specific strength, modulus, design flexibility, fatigue, and corrosion resistance [1,2]. Holes in aerospace composite structures, often for bolting or laying pipes and cables, can cause initial damage during mechanical drilling [3,4,5,6,7,8], such as peeling, delamination, tearing, raw edges, and fiber extraction, with delamination being the most common [9,10,11]. Drilling damage, including delamination, fiber separation, intra-layer cracking, and thermal degradation, is directly related to the drilling process and significantly reduces the residual load-bearing capacity of open-hole composites. Thus, studying drilling-induced delamination is crucial for understanding the structural integrity and performance of drilled composite components.

Drilling-induced delamination in composites has garnered significant research attention recently. This damage severely impacts the structural integrity of composites under various loads and notably reduces the residual strength of open-hole composites. Thus, understanding drilling-induced delamination is crucial for assessing the performance of drilled composite components.

In experiments, Karimi et al. [12] investigated how drilling-induced delamination affects the compressive properties of woven glass fiber-reinforced epoxy composites. They found that the delamination factor significantly impacts the material’s residual compressive strength. Zhuoyue et al. [13] studied the compressive behavior of open-hole laminates, discovering that cracks concentrate near hole edges. The damage process intensifies with more 45°/90° ply interfaces. Importantly, 45°/90° ply arrangements mitigate stress concentration and enhance load-bearing capacity. Zitoune et al. [14] explored the tensile behavior of composite plates with drilled and molded holes through experiments, shedding light on how these hole types impact the tensile properties of composites. Quantifying drilling-induced delamination in composite laminates is difficult, hindering damage tolerance assessments. To study how delamination location, size, and shape affect load-bearing capacity, artificially embedding delaminations is effective. Rhead et al. [15] explored delamination interactions and buckling mode transitions between layers. Nilsson et al. [16] used PTFE film to create delamination defects in composites. These studies show that artificial delamination is a useful tool for evaluating the mechanical properties and failure behavior of laminates with defects.

In numerical studies, Cui Yiyang et al. [17] applied deep learning to predict the tensile failure of composite plates with openings, highlighting deep learning’s potential for composite damage prediction and intelligent structural assessment. Zhengliang et al. [18] combined experiments and numerical methods to study damage evolution and failure in open-hole composite laminates under compression, developing a model that accounts for damage accumulation. Wysmulski [19] examined how open holes affect the buckling of compressed composite plates, showing the impact of hole position and size on buckling behavior.

This study addresses the gap in understanding the effects of multiple delamination defects on composites by focusing on two main aspects: (1) the compressive buckling and damage evolution in CFRP laminates with a single-delamination defect and (2) the compressive load-bearing capacity and failure characteristics of CFRP open-hole laminates with dual delamination defects. CFRP open-hole specimens with single pre-embedded circular delamination defects of varying sizes were fabricated. Damage propagation was characterized using C-scan, and in-plane deformation during loading was monitored with DIC. A cohesive zone-based numerical model was developed and calibrated against experimental results to accurately simulate damage propagation in single-defect laminates. The validated model was then used to predict and analyze the buckling, delamination propagation, and load-bearing capacity of CFRP open-hole laminates with dual delamination defects. This approach provides a comprehensive understanding of the material’s behavior under complex loading conditions, extending the knowledge from single-defect to multi-defect interactions.

## 2. Sample Preparation and Experiment

### 2.1. Sample Preparation

**Figure 1:**
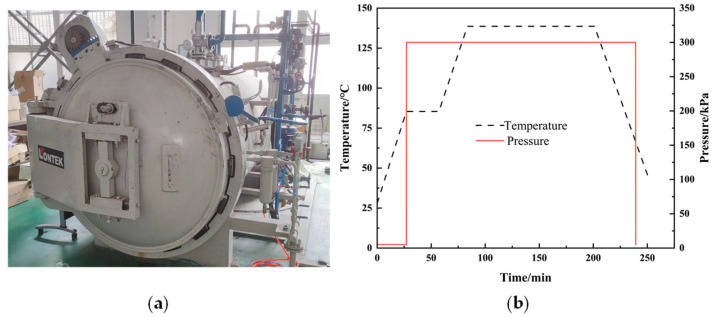
Composite curing equipment and curing conditions. (**a**) Schematic diagram of autoclave equipment. (**b**) Curing curve.

The raw materials used in this study were unidirectional carbon fiber prepregs (model HRC1-30%-A12-U-150gsm-1000-1) purchased from Hengshen Co., Ltd., Jiangsu Province. The composite laminates were fabricated using a hand lay-up and autoclave curing process. The curing steps are as follows: First, the autoclave temperature rose to 80 °C at 2 °C/min and stayed there for 30 min. Then, it went up to 130 °C at the same rate and was held for 120 min. Lastly, the autoclave’s water system cooled the chamber to under 50 °C at 2 °C/min. During heating and holding, the pressure was kept at 0.3 MPa. Figure 1 shows the autoclave and curing curves.

In preparation for fabrication, the dimensions of the composite samples were precisely defined as (*L*_1_ × *b* × *t*) 135 mm × 35 mm × 2.6 mm. The diameter of the circular hole (*d*) was set at 6 mm, and the gripping length at both ends (*L*_1_) was 30 mm. The diameters of the defect areas (*D*) were selected to be 10 mm, 15 mm, and 20 mm, respectively. Each prepreg layer had a thickness of 0.13 mm, and a total of 20 layers were used. The stacking sequence of the specimens was [45°/−45°/0°/0°/45°/−45°/0°/0°/0°/90°] s.

**Figure 2:**
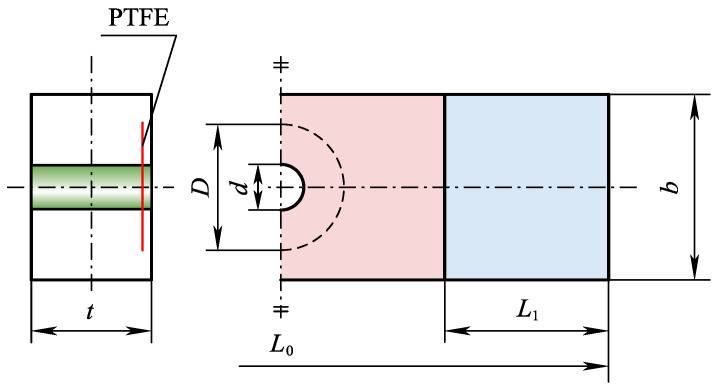
Schematic depiction of CFRP open-hole laminates with delamination.

During the layup process, a vacuum compression operation was performed every four layers of prepreg to eliminate residual bubbles between layers, thereby ensuring the structural integrity of the laminates. A PTFE film with a thickness of 0.1 mm was utilized to create a pre-embedded delamination defect, simulating initial delamination damage caused by mechanical drilling. A single pre-embedded delamination was placed between the second and third prepreg layers, with both the delamination defect and the hole positioned at the geometric center of the specimen, as shown in Figure 2.

For comparison, the study of defect-free CFRP open-hole laminates was also carried out and named Intact-0. The detailed dimensions of CFRP open-hole laminates with delamination defects are shown in Table 1.

**Figure 3:**
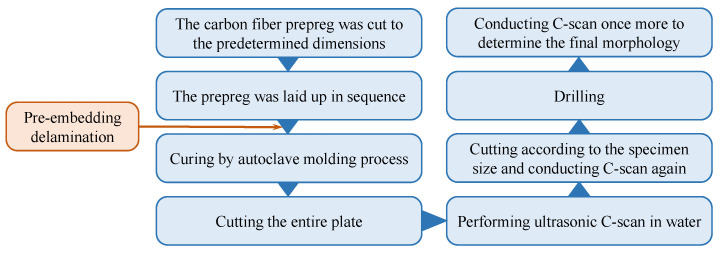
The preparation process of perforated carbon fiber composite plates with prefabricated layers.

The complete manual laying–curing–detection–cutting process is shown in Figure 3. After curing, the prepared composite laminates were tested by ultrasonic C-scan. After cutting, the specimens were tested by ultrasonic C-scan again to ensure that no additional damage was introduced by cutting.

### 2.2. Compression Test Scheme

The basic mechanical properties of unidirectional laminates were determined through tensile testing. The elastic modulus, Poisson’s ratio, and ultimate strength were measured in accordance with ASTMD3039 [20], ASTMD6641 [21], and ASTMD3518 [22] standards, respectively. The obtained material parameters are summarized in Table 1. They will be utilized for subsequent numerical analysis and predictions.

**Figure 4:**
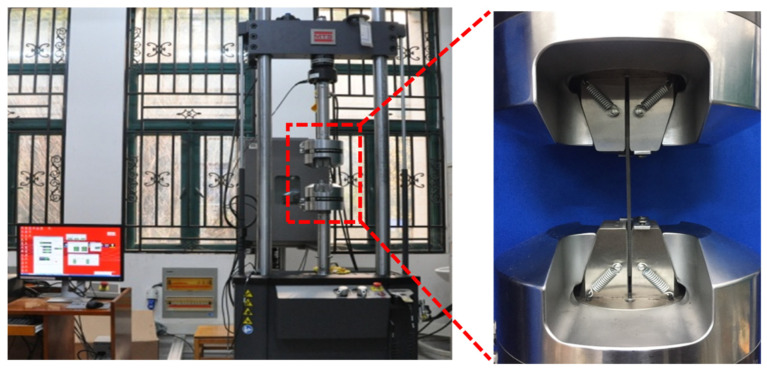
Schematic diagram of experimental device.

Compression tests were performed on defect-free and pre-embedded defect specimens using an MTS hydraulic servo universal testing machine from the United States under displacement control. A clamping fixture with a range of 0~7.2 mm was selected. The clamping length was 30 mm at each end of the specimen. The effective testing length was 75 mm in the middle. Specimens were vertically aligned with fixtures to prevent off-axis compression. Displacement–load curves were recorded. CFRP open-hole laminate specimens were tested at 0.05 mm/min, as shown in Figure 4.

### 2.3. Compression Test Result

**Figure 5:**
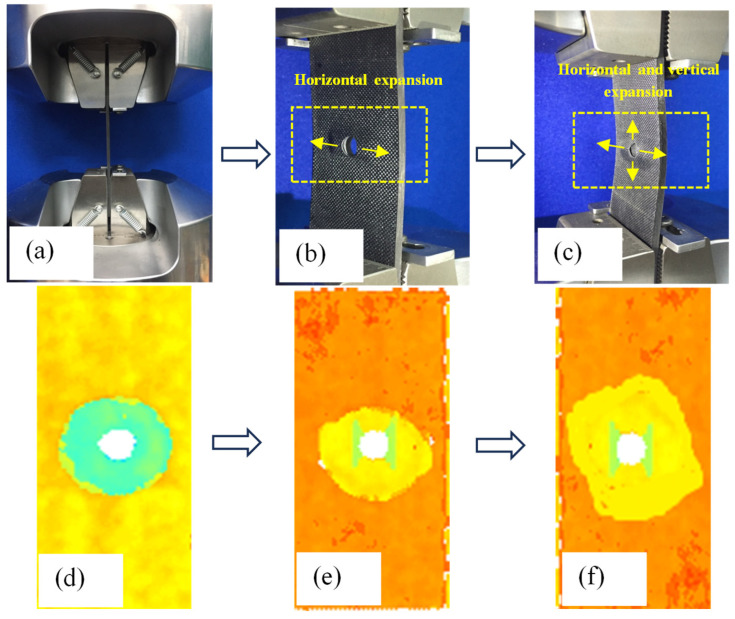
Buckling and lamination expansion of CFRP open-hole laminates with delamination. (**a**) Compression camping method. (**b**) Horizontal expansion. (**c**) Horizontal and vertical expansion. (**d**) C-scan inspection. (**e**) Horizontal expansion c-scan. (**f**) Horizontal and vertical expansion c-scan.

The behavior of CFRP laminates with a single pre-embedded delamination defect was studied. The analysis was based on experimental data and the comparison of buckling and progressive delamination processes during step loading. The behavior can be divided into three distinct stages, as shown in Figure 5.

(1) Linear Load-Bearing Stage: During the initial loading phase, no buckling occurs in the delaminated region. The displacement–load relationship exhibits linear growth, indicating stable structural load-bearing behavior.

(2) Local Buckling and Initial Delamination Propagation: As the displacement load increases, the specimen undergoes gradual local buckling deformation, reflected by a reduction in the slope of the displacement–load curve. When the load reaches a critical value, the thin laminate buckles, and delamination propagates horizontally (perpendicular to the loading direction). C-scan imaging reveals convex deformations in the upper and lower regions of the delamination zone, confirming the propagation behavior.

(3) Multi-Directional Delamination Propagation Stage: Upon further displacement loading, the main laminate participates in buckling. Delamination expansion becomes bidirectional, propagating both horizontally and vertically (parallel to the loading direction). Secondary C-scan detects irregular convex deformations on both sides of the delamination zone. The coordinated buckling of the sub-laminates and main laminates forms a “C”-shaped overall deformation.

**Figure 6:**
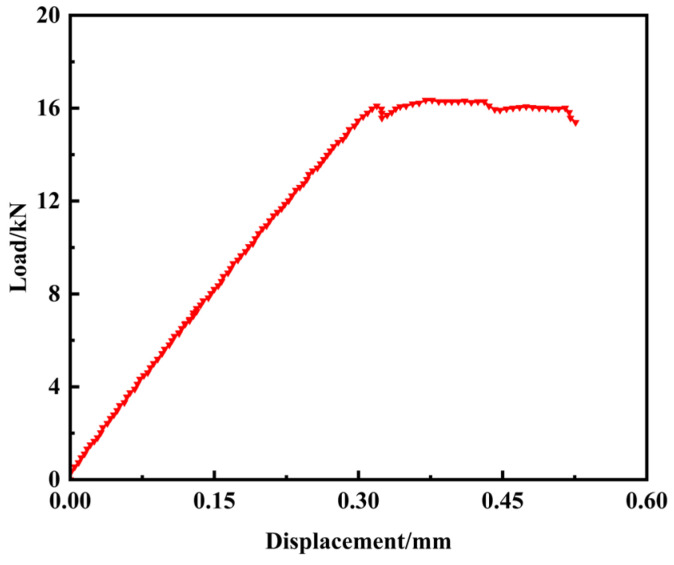
Load–displacement relationship in compressed CFRP laminates with open holes and delamination.

As shown in the displacement–load curve in Figure 6, the local critical buckling load for the DAMAGE-20 specimen is 16.17 kN. Delamination propagation initiates when the displacement load reaches approximately 0.3 mm. Following the onset of buckling-induced delamination, the curve gradually flattens, with each inflection point corresponding to a decrease in load-bearing capacity during delamination propagation.

**Figure 7:**
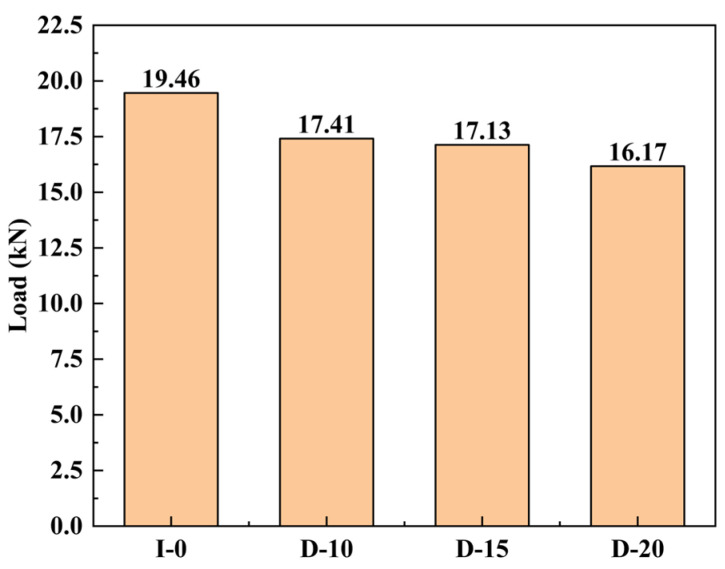
Failure load analysis of delaminated CFRP laminates with open holes under diverse working scenarios.

Figure 7 presents the relationship between compressive ultimate load and defect size of CFRP open-hole laminates containing a single pre-embedded delamination. The results demonstrate the following: (1) The defect-free open-hole CFRP laminate INTACT-0 exhibits an ultimate bearing capacity of 19.46 kN. (2) The introduction of delamination defects leads to a progressive reduction in ultimate load capacity with increasing defect area. The ultimate compressive loads for cases DAMAGE-10, DAMAGE-15, and DAMAGE-20 are 17.41 kN, 17.13 kN, and 16.17 kN, respectively, representing reductions of 10.5%, 12%, and 17%. (3) Larger defect areas intensify the coupling effect between delamination propagation and local buckling. The nonlinear reduction pattern reveals a gradual weakening rate (1.6% interval) at small defect sizes (DAMAGE-10 and DAMAGE-15), while a significantly accelerated reduction (5.6% interval) occurs at larger defect sizes (DAMAGE-20), indicating that delamination propagation accelerates structural failure more dramatically with larger defects.

## 3. Numerical Simulation of Open-Hole Composite Laminates with Delamination Defects

### 3.1. Numerical Model of Open-Hole Laminates with Delamination Defects

**Figure 8:**
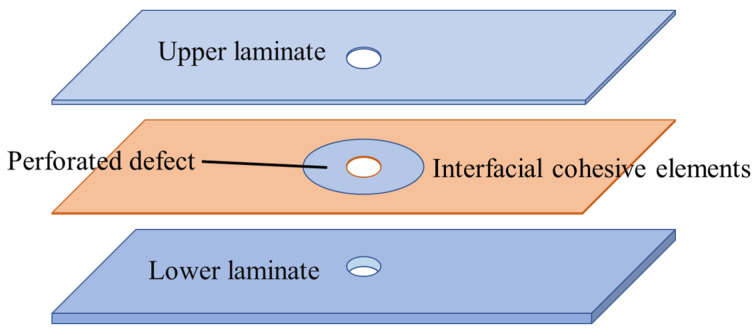
Finite element analysis framework for delaminated CFRP open-hole laminates.

**Figure 9:**
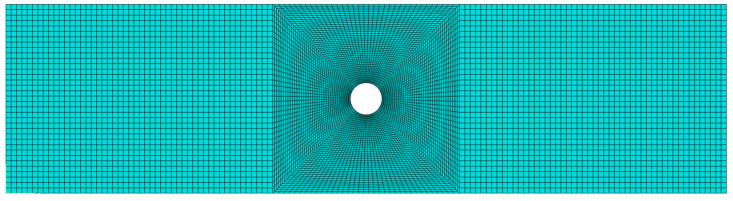
Finite element model meshing of CFRP open-hole laminates with delamination defects.

A finite element model for an open-hole CFRP laminate with delamination defects was created using Abaqus/Standard 2022. This software is proficient in managing complex scenarios like material plasticity, large deformations, and contact friction, and can precisely depict the anisotropy, nonlinearity, and damage evolution of composites. It thus provides a realistic simulation of composite mechanical behavior. Simulations were performed to study the buckling and delamination propagation under compression. The model includes an upper laminate, interfacial cohesive elements, and a lower laminate (Figure 8), with local mesh refinement near the defects (Figure 9). By specifying material properties, layup sequences, cohesive elements, and damage criteria, the model effectively simulated the damage initiation and evolution in the delaminated composite [23,24].

### 3.2. Cohesive Model

**Figure 10:**
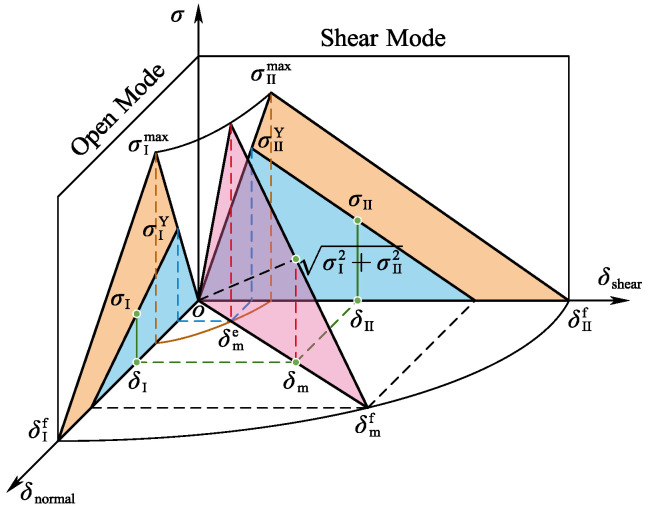
Bilinear mixed-mode softening law.

Delamination was modeled using zero-thickness cohesive elements with a bilinear constitutive law, as shown in Figure 10. Damage initiation was assessed via a quadratic nominal stress criterion [25].

(1) $${\left(\frac{t_{n}}{t_{n}^{0}}\right)}^{2}+{\left(\frac{t_{s}}{t_{s}^{0}}\right)}^{2}+{\left(\frac{t_{t}}{t_{t}^{0}}\right)}^{2}=1$$

In the equation, $t_{n}$, $t_{s}$, $t_{t}$ represent the interlaminar normal stress and shear stress of the element under load, respectively, and $t_{n}^{0}$, $t_{s}^{0}$, and $t_{t}^{0}$ denote the normal strength and shear strengths in two orthogonal directions, respectively.

The final failure of delamination damage was determined using a power-law-based mixed-mode failure criterion:

(2) $${\left(\frac{G_{n}}{G_{n}^{C}}\right)}^{\alpha }+{\left(\frac{G_{s}}{G_{s}^{C}}\right)}^{\alpha }+{\left(\frac{G_{t}}{G_{t}^{C}}\right)}^{\alpha }=1$$

In the equation, $G_{n}$, $G_{s}$, and $G_{t}$ represent the strain energy release rates for Mode I, II, and III damages, respectively. The superscript C denotes the critical fracture strain energy for each mode, while α is the empirical exponent in the power law. When the mixed-mode energy release rate reaches $G_{c}$, the cohesive element undergoes complete failure. The state variable D is the multiplier of the element’s initial stiffness, which gradually reduces the stress to zero. The damage variable D ranges from 0 to 1 [26].

(3) $$D=\frac{\delta^{f}(\delta -\delta^{0})}{\delta (\delta^{f}-\delta^{0})}$$

Here, δ represents the maximum relative displacement at a given increment step, while $\delta^{0}$ and $\delta^{f}$ denote the initiation displacement and complete failure displacement.

### 3.3. Damage Criterion

The Hashin criterion is a strength theory for predicting composite laminate failure, categorizing it into four modes based on material stress state and fiber/matrix strength properties: fiber tensile/compression failure and matrix tensile/compression failure. Its specific expression is detailed in references [27,28,29].

Fiber tensile failure:

(4) $${\left(\frac{\sigma_{11}}{X_{T}}\right)}^{2}\geq 1$$

Fiber compression failure:

(5) $${\left(\frac{\sigma_{11}}{X_{C}}\right)}^{2}\geq 1$$

Matrix tensile failure:

(6) $${\left(\frac{\sigma_{22}}{Y_{T}}\right)}^{2}+{\left(\frac{\sigma_{12}}{S_{12}}\right)}^{2}+{\left(\frac{\sigma_{13}}{S_{13}}\right)}^{2}+{\left(\frac{\sigma_{23}}{S_{23}}\right)}^{2}\geq 1$$

Matrix compression failure:

(7) $${\left(\frac{\sigma_{22}}{2S_{23}}\right)}^{2}+{\left(\frac{\sigma_{12}}{S_{12}}\right)}^{2}+{\left(\frac{\sigma_{13}}{S_{13}}\right)}^{2}+{\left(\frac{\sigma_{23}}{S_{23}}\right)}^{2}+\left(\frac{\sigma_{22}}{Y_{C}}\right)\left[{\left(\frac{Y_{C}}{2S_{23}}\right)}^{2}-1\right]\geq 1$$

Here, $\sigma_{11}$ denotes the stress in the direction of the fiber; $\sigma_{22}$ and $\sigma_{33}$ denote the transverse stress; $\sigma_{12}$, $\sigma_{13}$, and $\sigma_{23}$ denote shear stress; $X_{C}$ and $X_{T}$ represent the compressive strength and tensile strength in the direction of the fiber; $Y_{C}$ and $Y_{T}$, respectively, represent the transverse compressive strength and transverse tensile strength; and $S_{12}$, $S_{13}$, and $S_{23}$, represent the shear strength in three directions. 

### 3.4. Numerical Analysis Results of Open-Hole Composite Laminates with Delamination Defects

#### 3.4.1. Analysis of Compressive Buckling Simulation Results

Table 2 contrasts the compressive failure loads of CFRP open-hole laminates from finite element analysis and experiments across different conditions. The findings are as follows: (1) For the defect-free CFRP open-hole laminate (Intact-0), the numerical simulation result is 1.28% higher than the experimental result. (2) For CFRP open-hole laminates with a single pre-embedded delamination defect, the experimental failure loads exceed those from numerical simulations. Specifically, the failure load in the Damage-10 experiment is 9.76% higher than the numerical result, the Damage-15 failure load is 9.81% higher, and the Damage-20 failure load is 6.0% higher. This discrepancy arises because the material parameters in the simulation model are derived from experimental measurements, which inherently have errors. These results confirm the accuracy of the numerical simulation method.

**Figure 11:**
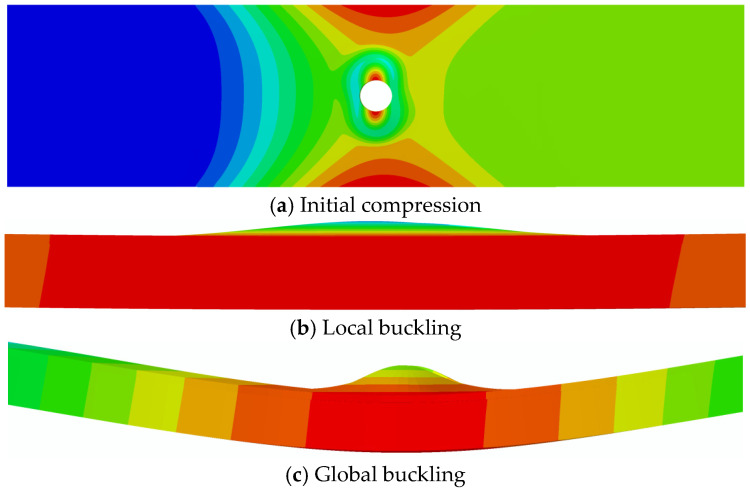
Deformation process of compressive buckling in open-hole laminates with delamination defects.

Figure 11 shows the deformation modes of open-hole composite laminates with delamination defects under compression. These modes, simulated using ABAQUS 2022, can be categorized into three primary stages: initial compression state, local buckling state, and global buckling state. In the figure, (a) depicts the initial compressive state of the model, where neither the upper nor lower laminates exhibit significant deformation; (b) shows that the thinner upper laminate has buckled, while the thicker lower laminate remains largely undeformed, representing the local buckling state of the entire mode; and (c) demonstrates significant deformation in both upper and lower laminates, with opposing displacement directions, indicating the global buckling state of the model. This deformation pattern corresponds well with the experimental results described earlier.

#### 3.4.2. Analysis of Compressive Delamination Propagation Simulation Results

**Figure 12:**
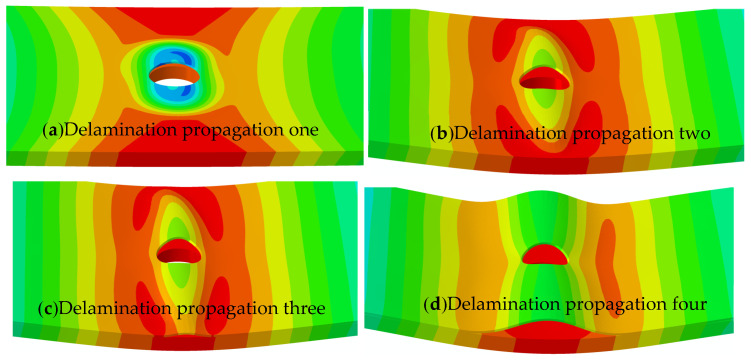
Compressive delamination propagation of open-hole composite laminates.

Figure 12 shows that the delamination propagation in open-hole composite laminates under compression matches earlier experimental observations. In the initial loading phase, compressive stresses trigger delamination initiation in the thinner upper laminate near the hole’s edge, as depicted in Figure 12a. As the load increases, the delamination spreads horizontally (perpendicular to the loading direction), as seen in Figure 12b,c. Upon further displacement loading, after complete widthwise propagation, the delamination begins to expand along the vertical direction (parallel to the loading direction), as presented in Figure 12d. These results confirm that the established buckling–delamination model with circular delamination accurately reproduces the experimental delamination growth behavior.

**Figure 13:**
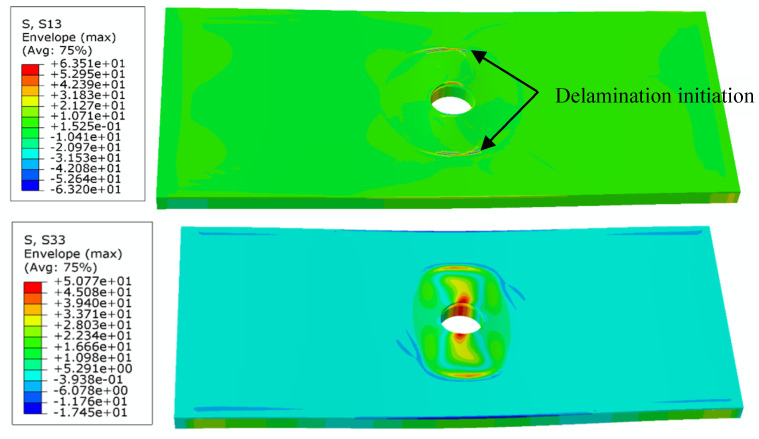
Buckling characteristics and initial delamination stress distribution in open-hole composite laminates.

The interfacial normal stress (σ_33_) and shear stress (σ_13_) during local buckling and initial delamination propagation in the pre-damaged region are extracted from the simulation and plotted in Figure 13. The results clearly indicate that delamination initiates at the hole edge due to significant stress concentration, where the maximum transverse stress (perpendicular to the loading direction) occurs. Consequently, the delamination preferentially propagates along the specimen’s width direction. Critical stress analysis reveals that the initiating shear stress σ_13_ (63.5 MPa) exceeds the Mode-I interlaminar shear strength *T*_I_, while the peel stress σ_33_ (50.8 MPa) remains below the Mode-II shear strength *T*_II_. This fundamental stress–strength relationship confirms that Mode-I fracture governs the initial delamination propagation phase.

**Figure 14:**
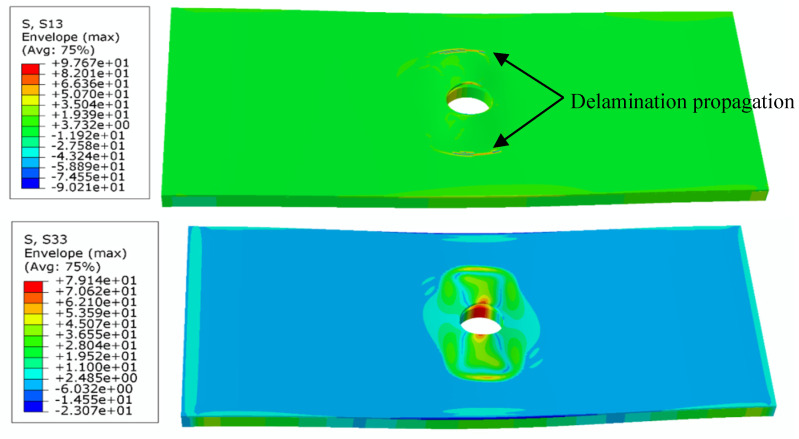
Stress contours of buckling and delamination propagation of open-hole composite laminates.

With continued loading, the contour plots of shear stress σ_13_ and peel stress σ_33_ during delamination propagation are obtained as shown in Figure 14. At this stage, the shear stress σ_13_ reaches 97.7 MPa, exceeding the Mode-I interlaminar shear strength (*T*_I_), while the peel stress σ_33_ attains 79.1 MPa, surpassing the Mode-II shear strength (*T*_II_). This stress state demonstrates that the delamination progresses under mixed-mode fracture conditions, ultimately leading to complete structural failure.

## 4. Dual-Defect Coupling Effects of Open-Hole Composite Laminates

In engineering applications, composite structures are prone to developing multiple through-thickness delamination defects. These defects are primarily caused by temperature gradients and residual stresses generated during the curing process. The interaction of these defects has a twofold effect. On one hand, it exacerbates the buckling susceptibility of laminates under compressive loads. On the other hand, it complicates the behavior of structural stiffness degradation. As a result, studying the buckling response of composite systems with multiple defects is crucial for guaranteeing the reliability of aerospace structures.

**Figure 15:**
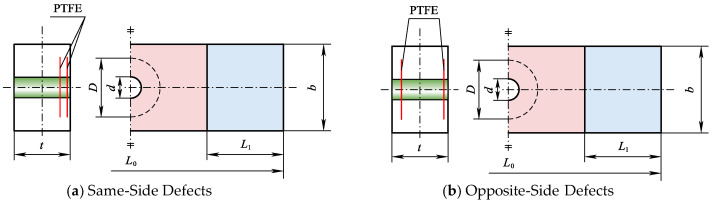
Schematic depiction of CFRP open-hole laminates with coupling defects.

Within the ABAQUS/Standard computational module, we have developed models of open-hole composite laminates with dual circular delamination flaws (20 mm in diameter). The models encompass two configurations: one with defects on the same side of the neutral plane, and another with defects on the opposite side of the neutral plane. The geometric schematics, defined parametrically, are detailed in Figure 15.

This study provides a systematic investigation into the compressive buckling characteristics and delamination propagation patterns in CFRP laminates with these two distinct defect configurations. It ultimately aims to clarify the mechanisms by which the spatial distribution of delamination affects structural stability.

### 4.1. Same-Side Defect Coupling Effects on Compressive Failure of Open-Hole Composite Laminates

**Figure 16:**
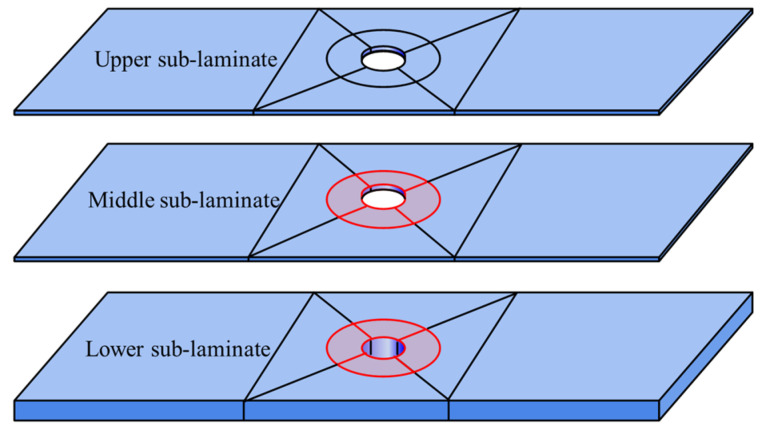
Finite element analysis framework for open-hole composite laminates with same-side defects.

This study focuses on open-hole composite laminates with the same-side defect. Artificial interlaminar defects were created between the second/third layers and the fourth/fifth layers, respectively. These defect interfaces partition the laminate into three distinct sub-laminates along the thickness direction: upper sub-laminate, middle sub-laminate, and lower sub-laminate. The schematic configuration is presented in Figure 16.

**Figure 17:**
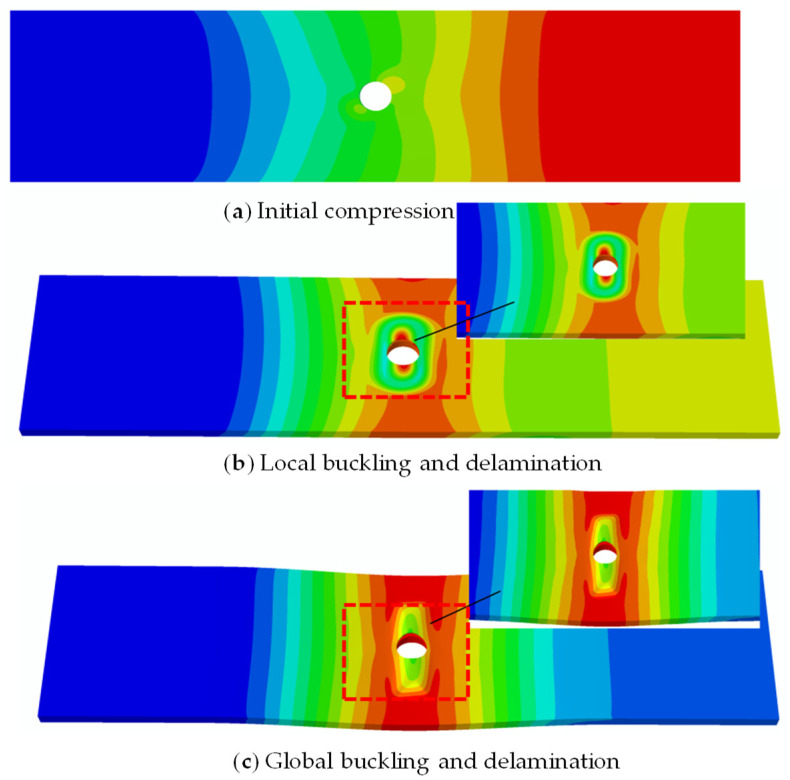
Compressive buckling and delamination propagation process in open-hole composite laminates with same-side defects.

**Figure 18:**
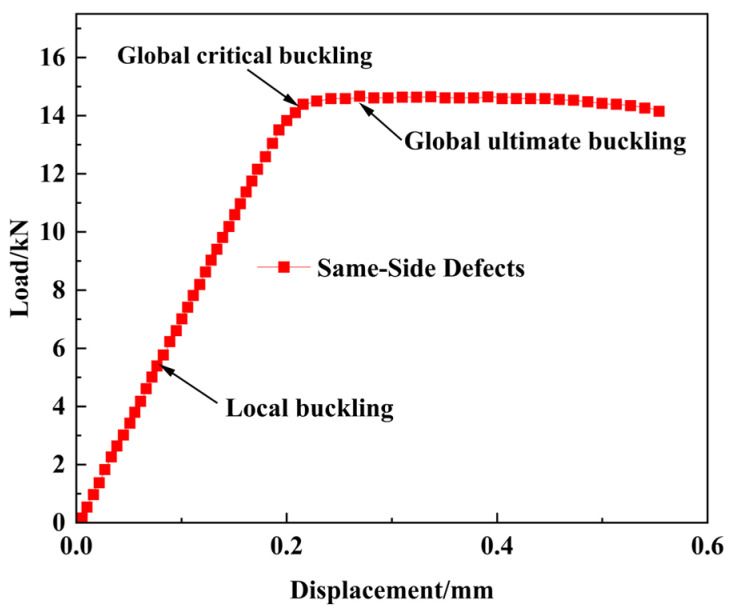
Displacement–load characteristics of open-hole composite laminates with same-side defects under compressive load.

As illustrated in Figure 17, the buckling behavior of the open-hole composite laminated structure with the same-side double defects also adheres to a three-stage process (initial compression, local buckling, and global buckling), which is similar to the single-delamination case. Figure 18 shows that in the same-side double defect configuration, the upper sub-laminate closer to the surface initiates local buckling at 5.41 kN, while the middle and lower sub-laminates remain stable. Then, global buckling occurs when the load further increases to 14.67 kN. Moreover, the delamination propagation pattern in this scenario is the same as that in single-delamination specimens.

### 4.2. Opposite-Side Defect Coupling Effects on Compressive Failure of Open-Hole Composite Laminates

**Figure 19:**
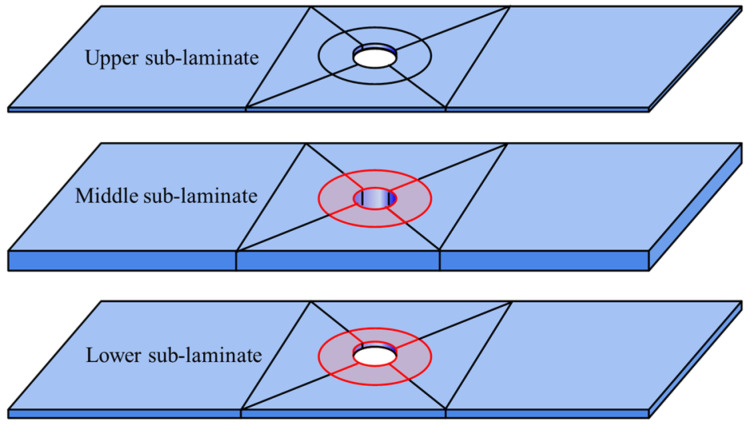
Finite element analysis framework for open-hole composite laminates with opposite-side defects.

This study investigates open-hole composite laminates with opposite-side double delamination defects. Artificial interlaminar defects were positioned between the 2nd/3rd layers and the 16th/17th layers. These defect interfaces partition the laminate into three sub-laminates along the thickness direction: the upper sub-laminate (between the top surface and first defect), the middle sub-laminate (between the two defects), and the lower sub-laminate (between the second defect and bottom surface). Figure 19 illustrates this configuration.

**Figure 20:**
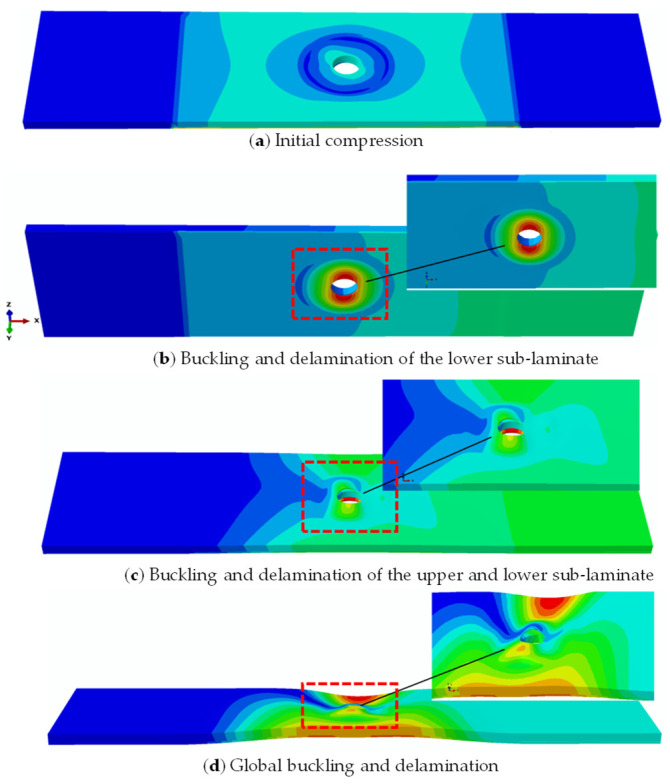
Compressive buckling and delamination propagation process in open-hole composite laminates with opposite-side defects.

**Figure 21:**
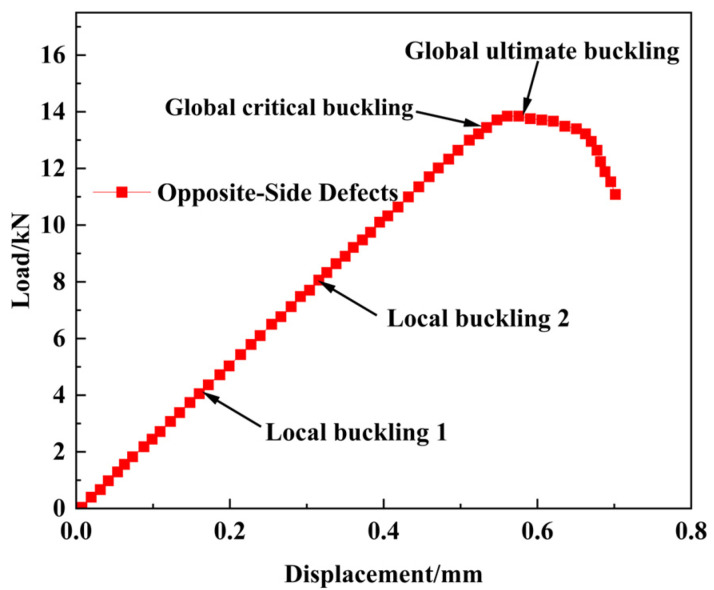
Displacement–load characteristics of open-hole composite laminates with opposite-side defects under compressive load.

As shown in Figure 20 and Figure 21, the buckling behavior of open-hole composite laminates with opposite-side double defects differs significantly from single-defect specimens. It exhibits a distinct three-stage failure process: (1) at 4.03 kN, local buckling of the lower sub-laminate (near the neutral plane) is initiated, accompanied by interfacial delamination propagation; (2) when the load reaches 7.94 kN, the upper sub-laminate (near the surface) begins local buckling; and (3) final global buckling occurs at 13.66 kN, leading to structural collapse. Notably, the delamination growth patterns on both sides of the neutral plane mirror those observed in single-circular-delamination specimens.

## 5. Conclusions

This study explores the compression buckling and delamination damage evolution of open-hole CFRP laminates through experimental and numerical methods, focusing on initial delamination damage caused by mechanical open holes. Key findings include the following:

(1) Delamination defects notably reduce the compressive strength of CFRP open-hole laminates. Specimens with a single-circular-delamination defect show a failure sequence of initial compression, local buckling, and global buckling. Delamination propagates along the specimen’s width first, then its length, leading to failure.

(2) The size of a single-circular-delamination defect significantly impacts the compressive strength of CFRP laminates, with larger defects causing greater reductions.

(3) CFRP laminates with dual delamination defects on the same side of the neutral plane fail similarly to those with a single defect. However, laminates with dual defects on opposite sides of the neutral plane exhibit a more complex failure mode. The sub-laminate closer to the neutral plane buckles locally first, followed by the opposite side. The interaction between the two defects complicates the failure process and significantly reduces compressive strength.

## Figures and Tables

**Table 1:** Mechanical properties and cohesive parameters of CFRP prepreg.

Parameter	Characteristic	Value
Longitudinal elastic modulus/MPa	*E*_11_	144,700
Transverse elastic modulus/MPa	*E*_22_ = *E*_33_	9650
In-plane modulus/MPa	*G*_12_ = *G*_13_	5800
Out-of-plane modulus/MPa	*G*_23_	4800
Poisson’s ratio	*υ*_12_ = *υ*_13_	0.3
Poisson’s ratio	*υ*_23_	0.45
Strain energy release rate/Jm^−2^	*G*_IC_	75
Strain energy release rate/Jm^−2^	*G*_II__C_	547
Fracture mode **I** intensity/MPa	*T*_I_	61
Fracture mode **II** intensity/MPa	*T*_II_	68
Interface stiffness/Nmm^−3^	*K*_P_	10^6^
B-K criterion power parameter	η	1.45

**Table 2:** Experimental and numerical analysis of compressive failure load for CFRP laminates with open holes and delamination defects under multiple conditions.

Type	Experimental Result (kN)	Numerical Simulation Result (kN)	Error
Intact-0	19.46	19.71	1.28%
Damage-10	17.41	15.71	9.76%
Damage-15	17.13	15.45	9.81%
Damage-20	16.17	15.20	6.0%

## Data Availability

The original contributions presented in this study are included in the article. Further inquiries can be directed to the corresponding authors.
